# Development of Self-Compressing BLSOM for Comprehensive Analysis of Big Sequence Data

**DOI:** 10.1155/2015/506052

**Published:** 2015-10-01

**Authors:** Akihito Kikuchi, Toshimichi Ikemura, Takashi Abe

**Affiliations:** ^1^Graduate School of Science and Technology, Niigata University, Niigata-shi, Niigata-ken 950-2181, Japan; ^2^Nagahama Institute of Bio-Science and Technology, Nagahama-shi, Shiga-ken 526-0829, Japan

## Abstract

With the remarkable increase in genomic sequence data from various organisms, novel tools are needed for comprehensive analyses of available big sequence data. We previously developed a Batch-Learning Self-Organizing Map (BLSOM), which can cluster genomic fragment sequences according to phylotype solely dependent on oligonucleotide composition and applied to genome and metagenomic studies. BLSOM is suitable for high-performance parallel-computing and can analyze big data simultaneously, but a large-scale BLSOM needs a large computational resource. We have developed Self-Compressing BLSOM (SC-BLSOM) for reduction of computation time, which allows us to carry out comprehensive analysis of big sequence data without the use of high-performance supercomputers. The strategy of SC-BLSOM is to hierarchically construct BLSOMs according to data class, such as phylotype. The first-layer BLSOM was constructed with each of the divided input data pieces that represents the data subclass, such as phylotype division, resulting in compression of the number of data pieces. The second BLSOM was constructed with a total of weight vectors obtained in the first-layer BLSOMs. We compared SC-BLSOM with the conventional BLSOM by analyzing bacterial genome sequences. SC-BLSOM could be constructed faster than BLSOM and cluster the sequences according to phylotype with high accuracy, showing the method's suitability for efficient knowledge discovery from big sequence data.

## 1. Introduction

As genome sequencing technologies represented by next-generation sequencers provide higher throughput performance, genome analyses of a very wide range of organisms can be conducted, and genomic sequence data have increased exponentially, resulting in a huge amount of data compiled in international nucleotide sequence databases (DDBJ/ENA/GenBank), which may soon reach the petascale. To enable efficient knowledge discovery from such big sequence data, it is important to examine the data comprehensively for clarifying the whole picture of all genome data available. This is especially important for phylogenetic classification of huge quantity of metagenomic data, which should contain a wide variety of novel prokaryotic and eukaryotic genomes poorly characterized previously.

We have focused on oligonucleotide composition in genomic sequences and developed a “Batch-Learning Self-Organizing Map (BLSOM),” which allows us to panoramically grasp the sequence characteristics unique to organism species by analyzing an ultralarge amount of genomic sequences. We have so far applied it to gene, genome, and metagenome analyses [1–3]. The method provides a strong clustering ability, with its result easily visible, under which sequences of genome fragments for each species are separated (“self-organized”) with high accuracy, based only on similarities in oligonucleotide composition, with absolutely no information given on the species in the course of computation. Furthermore, the algorithm can be optimized for parallel computations, enabling ultra-large-scale analyses performed by supercomputers, such as the “Earth Simulator” [4]. As BLSOM takes computation time proportional to approximately the cube of the quantity of input data, a large-scale BLSOM requires huge amounts of computational time and resources. With the appearance of next-generation sequencers, which has prompted genomic sequence data to grow at an exponential rate, enhancing computer performance alone (e.g., use of the new Earth Simulator) will not suffice and a higher-speed, larger-scale analysis strategy is now called for.

In this study, we have developed the “Self-Compressing BLSOM (SC-BLSOM),” which provides higher-speed computation and better clustering performance than the conventional BLSOM for species-known genomic sequences. The SC-BLSOM achieves higher speed by dividing input data into phylogenetic subclasses and structuring BLSOMs in a hierarchical manner. As mentioned before, the conventional BLSOM can cluster genomic sequences according to phylotypes, based only on similarities in oligonucleotide composition. Therefore, this unsupervised learning method can phylogenetically classify genomic sequences even derived from a novel gene, for which an orthologous sequence set required for constructing a reliable phylogenetic tree covering a wide range of phylotypes is not available. This shows the usefulness of BLSOM for phylogenetic classification of metagenomic sequences containing a large amount of novel genes and genetic fragments. In more detail, the conventional method of phylogenetic classification of metagenomic sequences in our previous studies [3–6] was the mapping of metagenomic sequences on a large-scale BLSOM, in which genomic fragments from almost all species-known genomes were classified (self-organized) according to phylotype in advance. When constructing this large-scale BLSOM with species-known sequences, addition of phylotype information of the sequences done in SC-BLSOM should most likely increase but should not decrease the phylogenetic clustering power for these sequences. Hence, SC-BLSOM is thought to be a kind of hybrid method of supervised and unsupervised learning because phylotype information is added in dividing the species-known genomic sequences into subclasses. We have tested the effectiveness of the SC-BLSOM by means of comparative studies of its computation time and clustering performance, by analyzing almost all prokaryotic genome sequences currently available.

## 2. Material and Method

### 2.1. Genome Sequence

Nucleotide sequences were obtained from http://www.ncbi.nlm.nih.gov/Genbank/. When the number of undetermined nucleotides (Ns) in a sequence exceeded 10% of the window size, the sequence was omitted from the analysis. When the number of Ns was less than 10%, the oligonucleotide frequencies were normalized to the length without Ns and included in the analysis.

### 2.2. Batch-Learning Self-Organizing Map (BLSOM)

Multivariate analyses such as factor corresponding analysis and principal component analysis (PCA) have been used successfully to investigate variations in gene sequences [7]. However, the clustering powers of conventional multivariate analyses are inadequate when massive quantities of sequence data from a wide variety of genomes are analyzed collectively. SOM implements nonlinear projection of multidimensional data onto a two-dimensional array of weight vectors, and this effectively preserves the topology of the high-dimensional data space [8–10]. We modified the conventional SOM for genome informatics on the basis of Batch-Learning SOM (BLSOM) to make the learning process and resulting map independent of the order of data input [1, 2]. The initial weight vectors were defined by PCA instead of random values on the basis of the finding that PCA can classify gene sequences into groups of known biological categories. Weight vectors (**w**_*ij*_) were arranged in the two-dimensional lattice denoted by *i* ( = 0,1,…, *I* − 1) and *j* ( = 0,1,…, *J* − 1). Weight vectors (**w**_*ij*_) were set and updated as described previously. A BLSOM program suitable for PC cluster systems and a PC program for mapping of new sequences on a large-scale BLSOM constructed with high-performance supercomputers can be obtained from our web site (http://bioinfo.ie.niigata-u.ac.jp/?BLSOM).

### 2.3. Self-Compressing BLSOM (SC-BLSOM)

**Figure 1:**
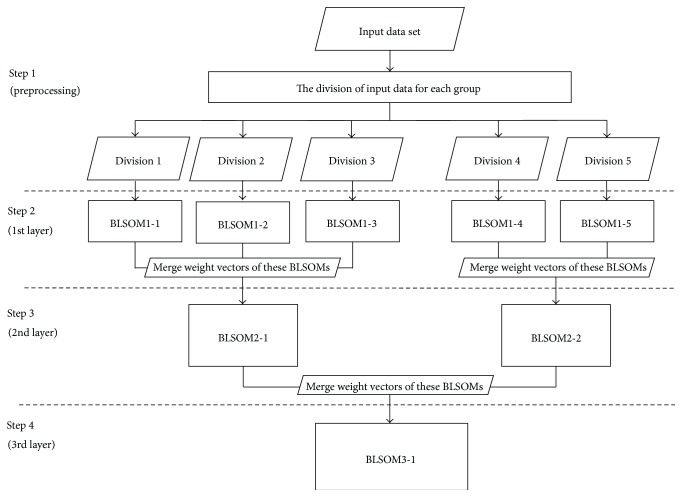
Overview of SC-BLSOM algorithm.

A conventional BLSOM performs clustering by means of reflecting the characteristics of input data onto the weight vectors, which are arranged on a two-dimensional lattice in the same format as the input data; that is, characteristics of the input data are summarized and compressed into weight vectors. The SC-BLSOM is an analytical method that fully takes advantage of the BLSOM, and Figure 1 shows the algorithm of the SC-BLSOM.

In Step 1, input data are divided according to data classification criteria “phylotype,” addition of phylogenetic information. As we use the genome sequence data of known prokaryotes for input data in this study, phylogenetic affiliation of the known prokaryote was used as the first-layer classification: Divisions 1–5 in Figure 1. In Step 2, BLSOM analysis is conducted on each group of the divided input data: the first-layer BLSOM. The number of BLSOM nodes (lattice points) created in this step was determined to be half the number of the divided data pieces. Weight vectors obtained in the first-layer BLSOMs of the divided input data are merged, according to the second-layer classification criterion representing a higher-order phylogenetic affiliation. Weight vectors in BLSOM1-1, BLSOM1-2, and BLSOM1-3 are merged and used for constructing BLSOM2-1 in Step 3, and those in BLSOM1-4 and BLSOM1-5 are merged and used for BLSOM2-2 in Step 3. In Step 4, the BLSOM analyses are performed using the merged weight vectors in the second-layer BLSOMs (BLSOM2-1 and BLSOM2-2). Steps 2 and 3 are repeated for additional layers, where the original input data are subdivided into more divisions.

In the SC-BLSOM, more BLSOMs are constructed than in the conventional BLSOM, but they need much shorter computation time than the BLSOM for all data input at once, because the division of data in the first-layer BLSOM has significantly reduced the number of input data pieces for each divided BLSOM. As mentioned before, BLSOM takes computation time proportional to approximately the cube of the quantity of input data. Smaller number of vectorial data pieces in the second-layer BLSOM than the original amount of data also cut computation time. Hence, shorter computation time can be expected for the SC-BLSOM compared with the conventional BLSOM.

## 3. Result and Discussion

### 3.1. Performance Comparison between SC-BLSOM and BLSOM

To test the basic performance of SC-BLSOM, its computation time and clustering performance were measured and compared with those for the conventional BLSOM. This test used genomic sequence data obtained by randomly picking out genomic sequences of 10 Kb from 817 different complete genomes of prokaryotes and merging them until the sequence length was one-tenth of the original data; BLSOM and SC-BLSOM were constructed with a degenerated tetranucleotide composition in a window size of 5 Kb; the frequencies of pairs of complementary tetranucleotides (e.g., AAAC and GTTT) in each fragment were summed up and abbreviated as DegeTetra [3]. The numbers of lattice points for the conventional BLSOM and for the SC-BLSOM in two layers were each set to be 50% of the quantity of input data. Input data for the first layer of SC-BLSOM were divided into 20 divisions using the number of phyla for the analyzed organisms as the classification criterion. The number of input data pieces amounted to 90,998 sequence fragments; Supplementary Table S1 shows the numbers of genomes and sequence fragments for each phylum (see Supplementary Material available online at http://dx.doi.org/10.1155/2015/506052).

**Figure 2:**
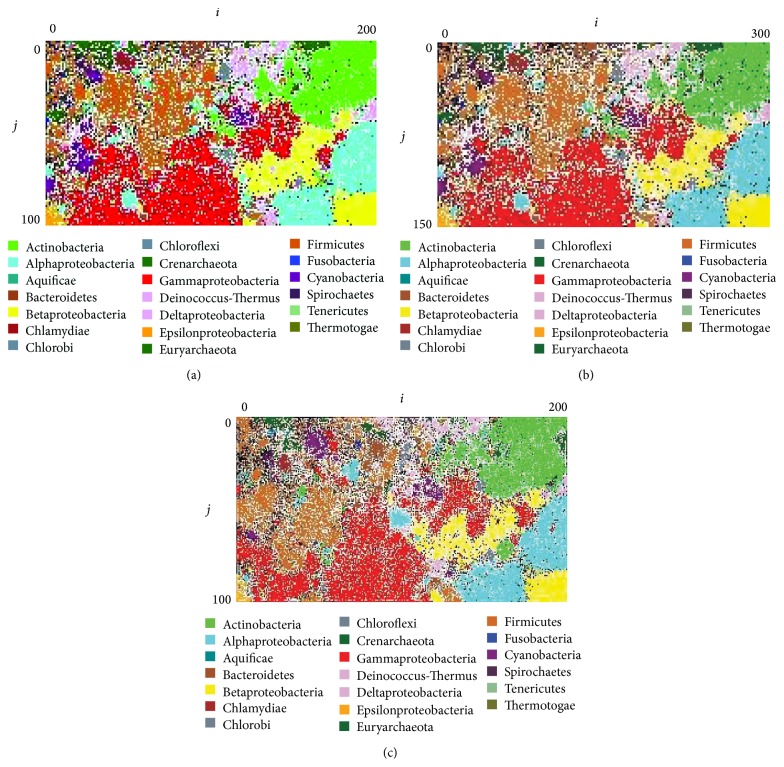
SC-BLSOM and BLSOM with DegeTetra in 5 Kb sequences from 817 microbial genomes. (a) SC-BLSOM. (b) BLSOM. (c) The original input data were mapped on SC-BLSOM. Lattice points that include sequences from more than one species are indicated in black, those that contain no genomic sequences are indicated in white, and those containing sequences from a single species are indicated in colors.

The analyses were conducted in a computer environment comprising Intel(R) Xeon(R) CPU E5-2680@2.70 GHz, 256 gigabytes of memory, and CentOS 5.11.  Figure 2 shows the classification results in the second layer of the SC-BLSOM and the conventional BLSOM; Supplementary Figure S1 shows the BLSOM maps created for each phylum in the first layer.  Table 1 shows the computation time and clustering performance of SC-BLSOM and the conventional BLSOM. Here, the clustering performance is presented as the percentage of the lattice points, on which only a single phylum was classified on the map.

The SC-BLSOM accomplished a reduced computation time approximately one-sixth of the time required for a conventional BLSOM, and as for the clustering performance the SC-BLSOM showed an improvement of about 3% over the conventional BLSOM. Additionally, in order to check whether the SC-BLSOM sufficiently reflected the characteristics of the original input data, the clustering performance was measured by plotting the original input data onto the second- layer SC-BLSOM (Figure 2(c)). As the SC-BLSOM reduces the map size in proportion to the decrease of input data, plotting the original input data produces approximately four data pieces per one node, resulting in a density twice that of the conventional BLSOM. Interestingly, the clustering performance was found to be better than that of the conventional BLSOM by 1% or more. This higher performance is thought to be benefitting from the addition of phylogenetic information for division conducted in Step 1. Accordingly, the SC-BLSOM can be described as a method, which is fast, capable of high clustering performance, and effective for constructing a large-scale BLSOM for species-known genomes; that is, the SC-BLSOM can be used as a reference map for phylogenetic assignment of a massive amount of metagenome sequences and thus be useful for phylogenetic characterization of an environmental ecosystem.

### 3.2. Test of Different First-Layer BLSOM Conditions

Computation time and clustering performance of the SC-BLSOM should be affected to a large degree by changing the number of weight vectors in the first-layer BLSOM. For this reason, we examined, in more detail, what the effects were from the quantity of weight vectors in the first-layer BLSOM. The number of weight vectors for the first SC-BLSOM was set to 25% of the quantity of input data, instead of 50% of the number of input data pieces used in Figure 2(a).

Table 2 shows that computation time is approximately two and a half hours for the SC-BLSOM, in which the number of weight vectors was set to 50% of the amount of input data and that the computation time is only twenty-one minutes for the SC-BLSOM, in which the number of weight vectors was set to 25%. While this SC-BLSOM had an evidently reduced computation time (approximately one-fortieth part of the conventional BLSOM), this condition exhibited lower clustering performance. Considering that the learning conditions for the second-layer BLSOM are the same as those for the SC-BLSOM shown in Figure 2(a), the performance degradation may be caused by the larger amount of information loss resulting from the decreased first-layer BLSOM map size (i.e., the decrease of the number of weight vectors relative to the quantity of input data). As a trial to overcome the reduction of clustering performance, we set a lower limit for the number of weight vectors in the first-layer BLSOM for maintaining adequate influence of the input data from small-size categories. This will bypass imbalances in the number of analyzed genomes in certain phyla used as the division category in Step 1; Supplementary Table S1 shows that many phyla have a limited number of sequence data pieces. When the lower limit was set at 30% of the amount of data assuming they are evenly divided, the total number of weight vectors of the first-layer BLSOMs increased, resulting in an increase in computation time of approximately one and a half times, and clustering performance improved. This shows the improvement effect on the data sets with small sample sizes.

Change in the condition for the first-layer BLSOM affects speed and clustering performance, showing a trade-off relationship between them. For example, if the aim is to quickly understand the whole picture of target species in a comparative genome analysis using all available genomes, a higher compression rate may be applied, as it suffices to grasp main features of each species. However, in estimating the biological phyla of metagenome sequences in detail, which should include sequences from a wide variety of unculturable species, BLSOMs with high clustering performance are required, and the aforementioned lower limit should be applied in order to get accurate clustering even for small-size data sets, which should be poorly represented in the current DNA databank. The level of clustering performance and computation speed should be set after taking into consideration the nature of the target data and the purpose of analysis.

### 3.3. Application of the SC-BLSOM for All Known Prokaryotes to Comparative Genome Analyses

To verify the actual performance of the SC-BLSOM for large quantities of genome sequence data, SC-BLSOM and BLSOM for all species-known prokaryote genomes currently available were constructed with DegeTetra composition; this covers a total of 3,500,000 5 kb sequences from 3,157 species, for which at least 10 kb of sequence was available from DDBJ/ENA/GenBank. By comparing the two BLSOMs, applicability of the SC-BLSOM to large-scale, comparative genome analyses was tested. In more detail, SC-BLSOM was arranged in three layers, with the data dividing criterion of family (divided into 301 divisions) for the first layer and phylum (divided into 38 divisions) for the second layer. The weight vectors were arranged so that they represent 50% of the quantity of input data. For computation of the SC-BLSOM, an Intel Xeon Phi 5110P (1.053 GHz, 60 cores) mounted-PC server was used. On the other hand, the conventional BLSOM was constructed using the Earth Simulator ES2, one of the leading supercomputer systems in Japan, to perform a parallel computation with 144 CPUs.

**Figure 3:**
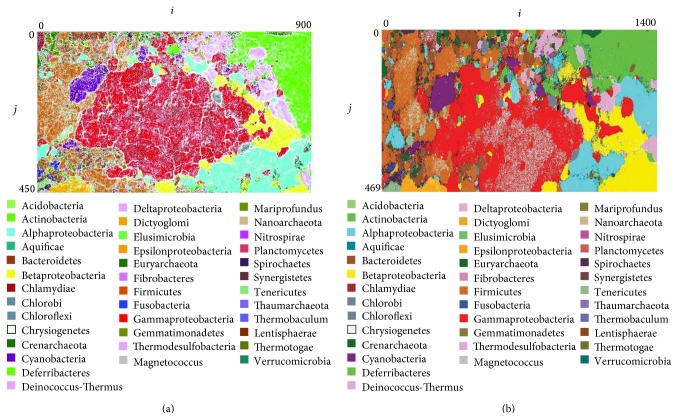
SC-BLSOM and BLSOM with DegeTetra in 5 Kb sequences from species-known prokaryotes. (a) SC-BLSOM. (b) BLSOM. Lattice points that include sequences from more than one species are indicated in black, those that contain no genomic sequences are indicated in white, and those containing sequences from a single species are indicated in colors.

Figure 3 shows the classification results from the phylum-based third-layer BLSOM in the SC-BLSOM (a), as well as that from the conventional BLSOM (b). The computation time was approximately one month for the conventional BLSOM, whereas it was approximately two weeks for the SC-BLSOM. While SC-BLSOM was performed using a normal PC server, a clustering performance was approximately 5% higher than that of the conventional BLSOM. We will briefly explain difference in clustering patterns between SC-BLSOM and BLSOM, by taking Gammaproteobacteria, for instance, which makes a large territory in the central part in the map. The number of white lattice points, which are devoid of genomic sequences in the final map, increases for SC-BLSOM, because the number of input sequences per lattice point for the third- layer map (a) was lower than that for the conversional BLSOM (b). Furthermore, continuous lines composed of white lattice points are observed. These while lines primarily correspond to borders between territories of different phylogenetic groups in Gammaproteobacteria and are formed because the detailed phylogenetic information has been included in the phylogenetic division for the first- and second-layer BLSOM: 155 and 35 divisions in Gammaproteobacteria, respectively.

**Figure 4:**
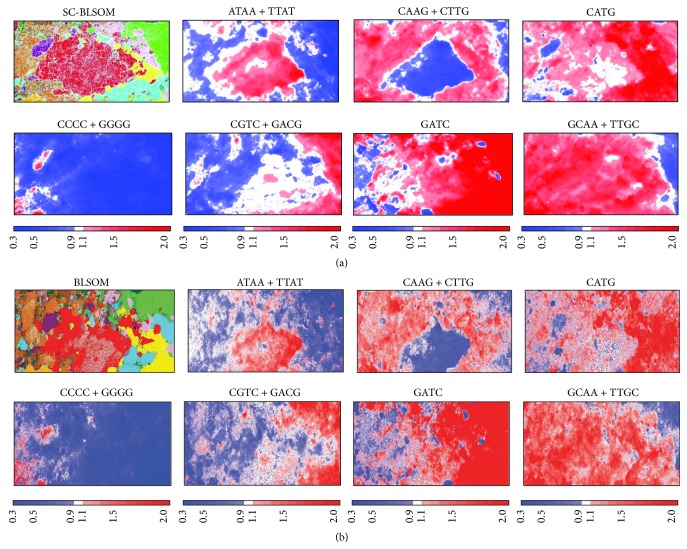
Level of each DegeTetra in SC-BLSOM (a) or BLSOM (b). Diagnostic examples of species-specific separations are presented. Level of each DegeTetra in each lattice point in SC-BLSOM or BLSOM in Figure 3 was calculated and normalized with the level expected from the mononucleotide composition of the lattice point. The observed/expected ratio is indicated in colors shown at the bottom of the figure.

Comparative genome analyses using the conventional BLSOM for oligonucleotide composition can reveal which oligonucleotides contribute to the formation of species- and phylotype-specific clusters, providing profound information about molecular evolutionary mechanisms establishing the species-specific oligonucleotide composition “genome signature” and possible biological functions of individual oligonucleotides [2, 11–16]. To examine whether the SC-BLSOM can be used to perform the same analysis as the conventional BLSOM, the distribution of oligonucleotide composition on SC-BLSOM was examined (Figure 4(a)). The level of each tetranucleotide for each reference weight vector was calculated and normalized with the level expected from the mononucleotide composition for the reference vector. Degenerated tetranucleotides diagnostic for phylum separations in the SC-BLSOM and conventional BLSOM are presented in Figure 4. ATAA + TTAT (a pair of ATAA and TTAT) is characteristically overrepresented in the Gammaproteobacteria territory. CAAG + CTTG is underrepresented in the Gammaproteobacteria territory. In Cyanobacteria territory, CCCC + GGGG is overrepresented, but CATG is overrepresented. These findings obtained in Figure 4 should not depend on the set of genomes included in the analysis because the level of each tetranucleotide for each lattice point was normalized with the level expected from the mononucleotide composition for the lattice point; the observed/expected ratio is represented with the level of red (overrepresented) or blue (underrepresented) color. SC-BLSOM and the conventional BLSOM give similar patterns, showing that the SC-BLSOM can also unveil species-specific (and phylotype-specific) characteristics of oligonucleotide composition, by visualizing a major combination of oligonucleotide frequencies contributing to sequence clustering (self-organization). Collectively, SC-BLSOM can be used for searching for hidden genome signatures and for large-scale comparative genome analyses, which will provide profound knowledge of individual genomes on evolutionary and functional aspects.

## 4. Conclusion

We have developed a Self-Compressing BLSOM (SC-BLSOM), which provides higher-speed and better clustering performance than the conventional BLSOM. This high-speed is achieved by dividing input data according to phylogenetic group and structuring the layered BLSOMs. Actual application to the comparative genome analyses of more than 3,000 prokaryotic genomes currently available demonstrated the method's effectiveness. The SC-BLSOM performs analyses in a layered manner, and, therefore, it performs faster as the data are divided further; the smaller number of data pieces in the first-layer BLSOM reduces the map size, resulting in shorter computation time. Furthermore, the SC-BLSOM can be easily performed in parallel using multiple computers without requiring special techniques, because the first-layer BLSOM processes are completely independent of one another.

The SC-BLSOM can be applied to both comparative genome analyses and phylogenetic estimation of metagenomic sequence [3, 17–22]. With conventional BLSOM, we published also a prediction of chronological, directional change in the influenza viral genome sequences during a pandemic [23]. In addition, the BLSOM for oligopeptide [24], as well as the oligopeptide SC-BLSOM, can be used for function prediction of poorly characterized proteins. Therefore, SC-BLSOM can analyze not only the genome sequence data which will undoubtedly grow to an ultralarge volume, but also a wide variety of multidimensional data pieces, for example, oligopeptide composition in proteins. Because of its powerful visualization ability and high-speed, SC-BLSOM will become a versatile tool for effective knowledge discovery from big data; that is, this method will become useful for a wide variety of classification purposes against big vectorial data pieces, not only in genomics but also in other fields.

## Supplementary Material

Supplementary data list the numbers of species and sequence fragments that were used in SC-BLSOM and conventional BLSOM, and the BLSOM created for each phylum in the first layer at Figure 2. Supplementary Table S1. Numbers of species and sequence fragments that were used in SC-BLSOM and BLSOM at Figure 2. Supplementary Figure S1. BLSOM with DegeTetra in 5-Kb sequences from each phylum in the first layer. (a) Actinobacteria, (b) Alphaproteobacteria, (c) Aquificae, (d) Bacteroidetes, (e) Betaproteobacteria, (f) Chlamydiae, (g) Chlorobi, (h) Chloroflexi, (i) Crenarchaeota, (j) Cyanobacteria, (k) Deinococcus-Thermus, (k) Deltaproteobacteria, (l) Epsilonproteobacteria, (m) Euryarchaeota, (n) Firmicutes, (o) Firmicutes, (p) Fusobacteria, (q) Gammaproteobacteria, (r) Spirochaetes, (s) Tenericutes, (t) Thermotogae.

## Figures and Tables

**Table 1:** Computational time and occurrence level of pure lattice points in SC-BLSOM and BLSOM.

	BLSOM	SC-BLSOM	SC-BLSOM (mapped original data set)
Computational time (min)	831	138	—
Occurrence level (%) (^*∗*^1)	93.5	96.0	94.5

^*∗*^1: occurrence level is the percentage of the lattice points on which only a single phylum was classified on the obtained map. Calculated using the formula: 100 × (number of lattice points on which there is only a single phylum)/(number of total lattice points).

**Table 2:** Computational time and occurrence level of pure lattice points in different compression rate of SC-BLSOM.

	SC-BLSOM	SC-BLSOM	SC-BLSOM defined lower limit
Compression rate (^*∗*^1)	2	4	2
Computational time (min)	138	21	176
Occurrence level (%) (^*∗*^2)	96.0	94.1	96.6

^*∗*^1: compression rate was defined by the number of input data pieces mapped on a weight vector.

^*∗*^2: occurrence level is the percentage of the lattice points on which only a single phylum was classified on the obtained map. Calculated using the formula: 100 × (number of lattice points on which there is only a single phylum)/(number of total lattice points).
