# Supplementary material for: Development of Self-Compressing BLSOM for Comprehensive Analysis of Big Sequence Data
Source: Biomed Res Int. 2015 Oct 1;2015:506052. doi: 10.1155/2015/506052 (PMC4606171; doi:10.1155/2015/506052)
Supplement: Supplementary file 1 — Supplementary data list the numbers of species and sequence fragments that were used in SC-BLSOM and conventional BLSOM, and the BLSOM created for each phylum in the first layer at Figure 2. Supplementary Table S1. Numbers of species and sequence fragments that were used in SC-BLSOM and BLSOM at Figure 2. Supplementary Figure S1. BLSOM with DegeTetra in 5-Kb sequences from each phylum in the first layer. (a) Actinobacteria, (b) Alphaproteobacteria, (c) Aquificae, (d) Bacteroidetes, (e) Betaproteobacteria, (f) Chlamydiae, (g) Chlorobi, (h) Chloroflexi, (i) Crenarchaeota, (j) Cyanobacteria, (k) Deinococcus-Thermus, (k) Deltaproteobacteria, (l) Epsilonproteobacteria, (m) Euryarchaeota, (n) Firmicutes, (o) Firmicutes, (p) Fusobacteria, (q) Gammaproteobacteria, (r) Spirochaetes, (s) Tenericutes, (t) Thermotogae. [file 506052.f1.pdf]

Supplementary Table S1. Numbers of Species and sequence fragments that were used in SC-BLSOM and BLSOM at Figure 2.

Phylum	Species#	sequence fragments#
Actinobacteria	91	10935
Alphaproteobacteria	103	9921
Aquificae	8	304
Bacteroidetes	31	2883
Betaproteobacteria	63	8557
Chlamydiae	8	622
Chlorobi	10	575
Chloroflexi	11	957
Crenarchaeota	23	1266
Cyanobacteria	13	2700
Deinococcus-Thermus	8	473
Deltaproteobacteria	32	3776
Epsilonproteobacteria	20	1724
Euryarchaeota	58	2823
Firmicutes	150	17219
Fusobacteria	5	255
Gammaproteobacteria	139	24067
Spirochaetes	17	1097
Tenericutes	16	407
Thermotogae	11	437
Total	817	90998

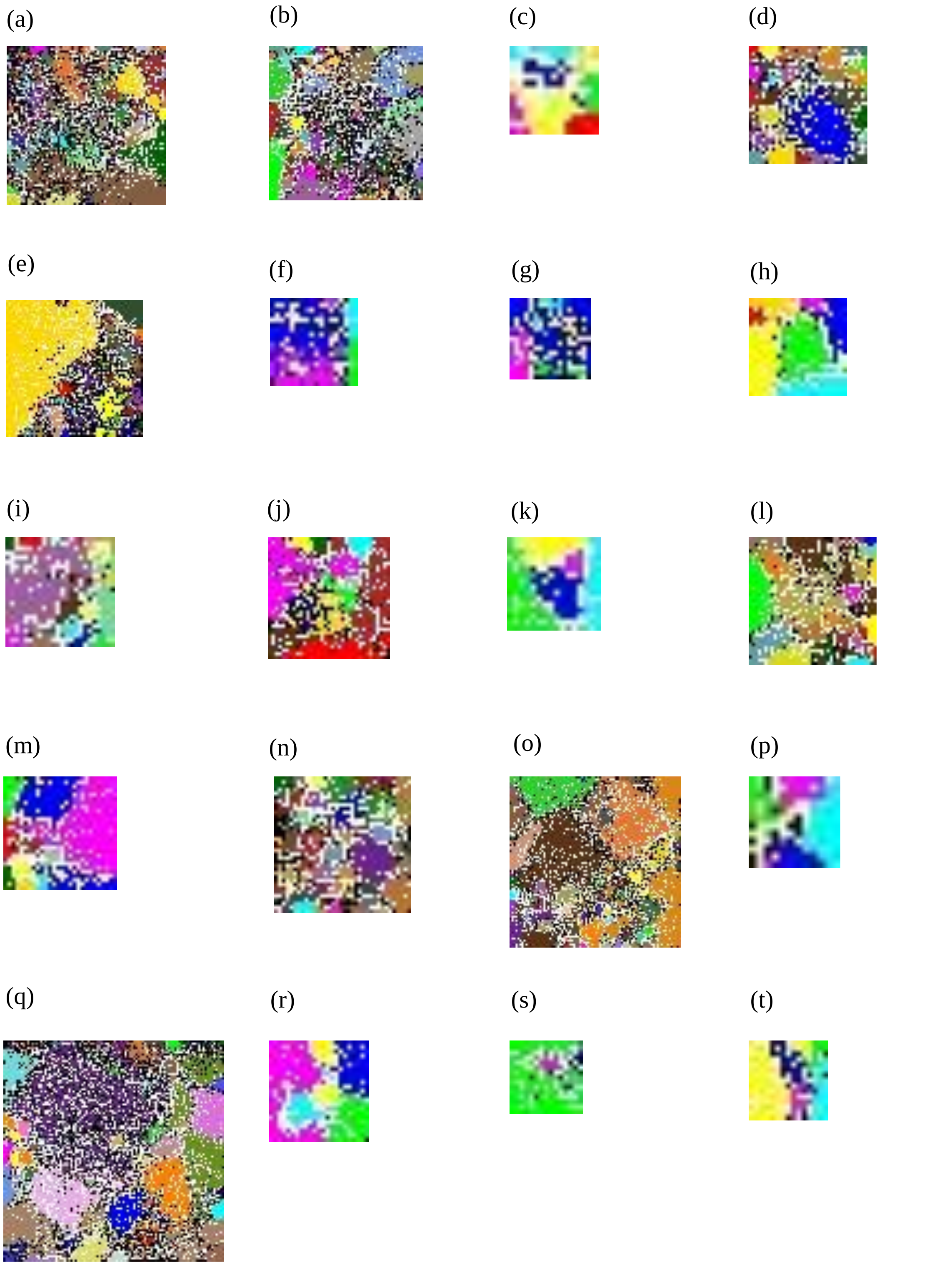

Supplementary Figure S1. BLSOM with DegeTetra in 5-Kb sequences from each phylum in the first layer. (a) Actinobacteria, (b) Alphaproteobacteria, (c) Aquificae, (d) Bacteroidetes, (e) Betaproteobacteria, (f) Chlamydiae, (g) Chlorobi, (h) Chloroflexi, (i) Crenarchaeota, (j) Cyanobacteria, (k) Deinococcus-Thermus, (k) Deltaproteobacteria, (l) Epsilonproteobacteria, (m) Euryarchaeota, (n) Firmicutes, (o) Firmicutes, (p) Fusobacteria, (q) Gammaproteobacteria, (r) Spirochaetes, (s) Tenericutes, (t) Thermotogae.
